# Peptide location fingerprinting identifies structural alterations within basement membrane components in ageing kidney

**DOI:** 10.1016/j.matbio.2023.07.001

**Published:** 2023-07-10

**Authors:** Alexander Eckersley, Mychel RPT Morais, Matiss Ozols, Rachel Lennon

**Affiliations:** a Division of Musculoskeletal & Dermatological Sciences, School of Biological Sciences, Faculty of Biology, Medicine and Health, The University of Manchester, Manchester, UK; b Division of Cell Matrix Biology & Regenerative Medicine, School of Biological Sciences, Faculty of Biology, Medicine and Health, The University of Manchester, Manchester, UK; c Wellcome Centre for Cell-Matrix Research, School of Biological Sciences, Faculty of Biology, Medicine and Health, The University of Manchester, Manchester, UK; d Department of Human Genetics, Wellcome Sanger Institute, Genome Campus, Hinxton, UK; e British Heart Foundation Centre of Research Excellence, University of Cambridge, Cambridge, UK; f Department of Paediatric Nephrology, Royal Manchester Children’s Hospital, Manchester University Hospitals NHS Foundation Trust, Manchester, UK

**Keywords:** Proteomics, Peptide location fingerprinting, Ageing, Basement membrane, Kidney, Glomerulus, Tubulointerstitium, Lung, Mass spectrometry, Human, Mouse

## Abstract

During ageing, the glomerular and tubular basement membranes (BM) of the kidney undergo a progressive decline in function that is underpinned by histological changes, including glomerulosclerosis and tubular interstitial fibrosis and atrophy. This BM-specific ageing is thought to result from damage accumulation to long-lived extracellular matrix (ECM) protein structures. Determining which BM proteins are susceptible to these structure-associated changes, and the possible mechanisms and downstream consequences, is critical to understand age-related kidney degeneration and to identify markers for therapeutic intervention. Peptide location fingerprinting (PLF) is an emerging proteomic mass spectrometry analysis technique capable of identifying ECM proteins with structure-associated differences that may occur by damage modifications in ageing. Here, we apply PLF as a bioinformatic screening tool to identify BM proteins with structure-associated differences between young and aged human glomerular and tubulointerstitial compartments. Several functional regions within key BM components displayed alterations in tryptic peptide yield, reflecting potential age-dependent shifts in molecular (e.g. laminin-binding regions in agrin) and cellular (e.g. integrin-binding regions in laminins 521 and 511) interactions, oxidation (e.g. collagen IV) and the fragmentation and release of matrikines (e.g. canstatin and endostatin from collagens IV and XVIII). Furthermore, we found that periostin and the collagen IV α2 chain exhibited structure-associated differences in ageing that were conserved between human kidney and previously analysed mouse lung, revealing BM components that harbour shared susceptibilities across species and organs.

## Introduction

Basement membranes (BM) are functional barriers composed of a meshwork of specialised extracellular matrix (ECM) proteins that underlie continuous layers of cells [1]. In the kidney, BMs play a unique role in the glomerulus, where they exist sandwiched between podocytes and endothelial cells, creating a blood filtration barrier which restricts the flow of molecules based on size and charge [2], and around the Bowman’s capsule and tubules. As in all BMs, including those in the kidney vasculature and tubules, the glomerular BM (GBM) consists of a laminin layer overlaid with a collagen IV layer, which are interconnected by the bridging proteins nidogen and perlecan. Uniquely however, the mature GBM is markedly thicker, being derived from the fusion of distinct endothelial and podocyte BMs during development, and it contains distinct isoforms of laminin (521) and collagen IV (α3α4α5). These molecules are crucial for filtration barrier function [2], with variants in *LAMB2* resulting in Pierson syndrome [3] and in *COL4A3, COL4A4* and *COL4A5* manifesting as Alport syndrome [4,5].

During ageing, the kidney undergoes a progressive decline in function [6] which can be characterised by a 5–10% fall in glomerular filtration rate per decade after the age of 35 years [7,8]. This deterioration is underlined by histological changes including GBM thickening [9] and glomerulosclerosis as well as tubular atrophy and tubulointerstitial (TBI) fibrosis, which can be further exacerbated in age-related chronic kidney disease [10]. Although the mechanisms behind these ageing processes remain elusive, BM remodelling is a key feature which likely relates directly to the decline in filtration function. Recently, we conducted a proteomic analysis of aged and young glomerular (GBM-enriched) and TBI (tubular BM-enriched) human kidney samples, in which we demonstrated a decrease in BM components, such as laminins, collagen IV and agrin and an increase in interstitial ECM, such as elastic fibre components, collagen V and aggrecan in aged, highlighting a dynamic shift in glomerular and tubular BM composition with age [11].

In contrast to intracellular proteins, ECM proteins are predominantly long-lived [12], with some components such as fibrillar collagens and elastic fibres remaining present throughout the human lifespan [13,14]. Similar observations have been made for GBM components in isotopically labelled mice, where several laminin and collagen IV chains were shown to have extremely low turnover [15]. As a result, these ECM proteins are thought to be susceptible to a unique form of ageing [16], where structural damage by mechanisms such as oxidation [17,18], glycation [19,20] and fragmentation [21,22] can be accumulated over time through chronic exposure to reactive oxygen species (ROS), sugars and matrix metalloproteinases (MMPs) respectively. As such, we proposed previously [11] that the changes in BM composition observed in ageing could result from damage accumulation to these long-lived ECM proteins, combined with a reduced cellular capacity to regenerate [23]. Determining which BM proteins are susceptible to structure-associated changes, and the mechanisms and downstream consequences, is critical to understand age-related kidney degeneration and to identify of markers for therapeutic intervention.

The generation of age-associated damage modifications to the higher order structure of BM proteins may manifest independently from changes to protein abundance and architecture, making them challenging to identify using traditional biochemical approaches. Peptide location fingerprinting (PLF) is an emerging proteomic analysis technique, capable of identifying ECM proteins with significant structure-associated differences that may occur because of damage modifications in ageing [24–27]. By mapping, quantifying and statistically testing tryptic peptides (identified by liquid chromatography tandem mass spectrometry [LC-MS/MS]) along protein structures, PLF takes advantage of the partial solubility of the ECM to detect, in an unbiassed manner, regional differences in peptide yield that may arise due to damage or changes in protein interactions or conformation. In addition to the direct detection of molecular-level damage *in vitro* which relate to changes in ECM higher order ultrastructure [24], PLF has been applied *in vivo* to identify: 1) biomarker candidates of photoageing in human skin and ageing in tendon [25], 2) tissue region-conserved structural differences within ageing, human intervertebral disc ECM proteins [26, 27], and 3) protein functional domains with specific structural differences in ageing and disease (atherosclerosis) that were shared between mouse and human [27]. Most promisingly, PLF can identify potential mechanisms and consequences of ageing [27], as previously shown in human intervertebral disc, where significant differences in the collagen I C-propeptide domain, and within a region downstream of a prominent MMP cleavage site, served as indicators of both diminished collagen synthesis and heightened fragmentation respectively [26].

**Fig. 1:**
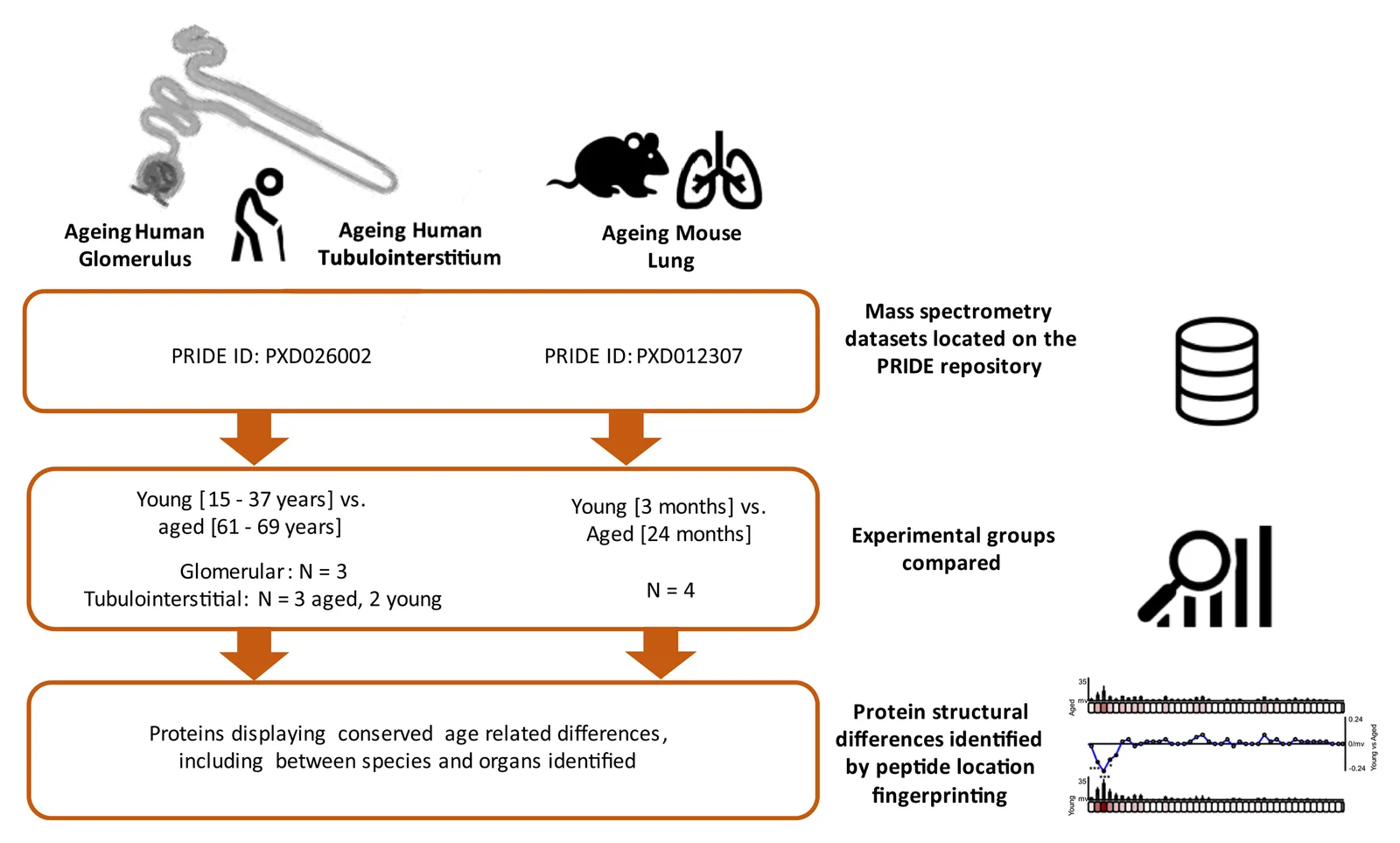
PLF analysis was used to identify BM proteins with structure-associated differences between aged and young human kidney glomerular and TBI LC-MS/MS datasets (available on PRIDE). These potential kidney markers were then further compared against those previously identified in mouse lung [27] to reveal BM components which may share the same mechanisms or consequences of ageing between both species and organs.

We recently published a proteomic LC-MS/MS analysis of young and aged human kidney glomerular and tubulointerstitial samples [11], but did not examine structure-associated differences to GBM and tubular BM proteins. This provided an opportunity to apply PLF, not only for the bioinformatic identification of novel biomarker candidates of ageing, but also to characterise the structural alteration of BM components which are likely to play important roles in the decline of kidney function. Additionally, we previously used PLF to interrogate ECM ageing in mouse lung [27]. Since lung alveolar and kidney glomerular BMs have similar composition with comparable filtration barrier functions [28], we also aimed to compare lung and kidney analyses to identify BM proteins which potentially harbour shared susceptibilities across different species and organs (Fig. 1).

## Results and discussion

### Glomerular basement membrane proteins undergo structure-specific alterations in ageing

**Fig. 2:**
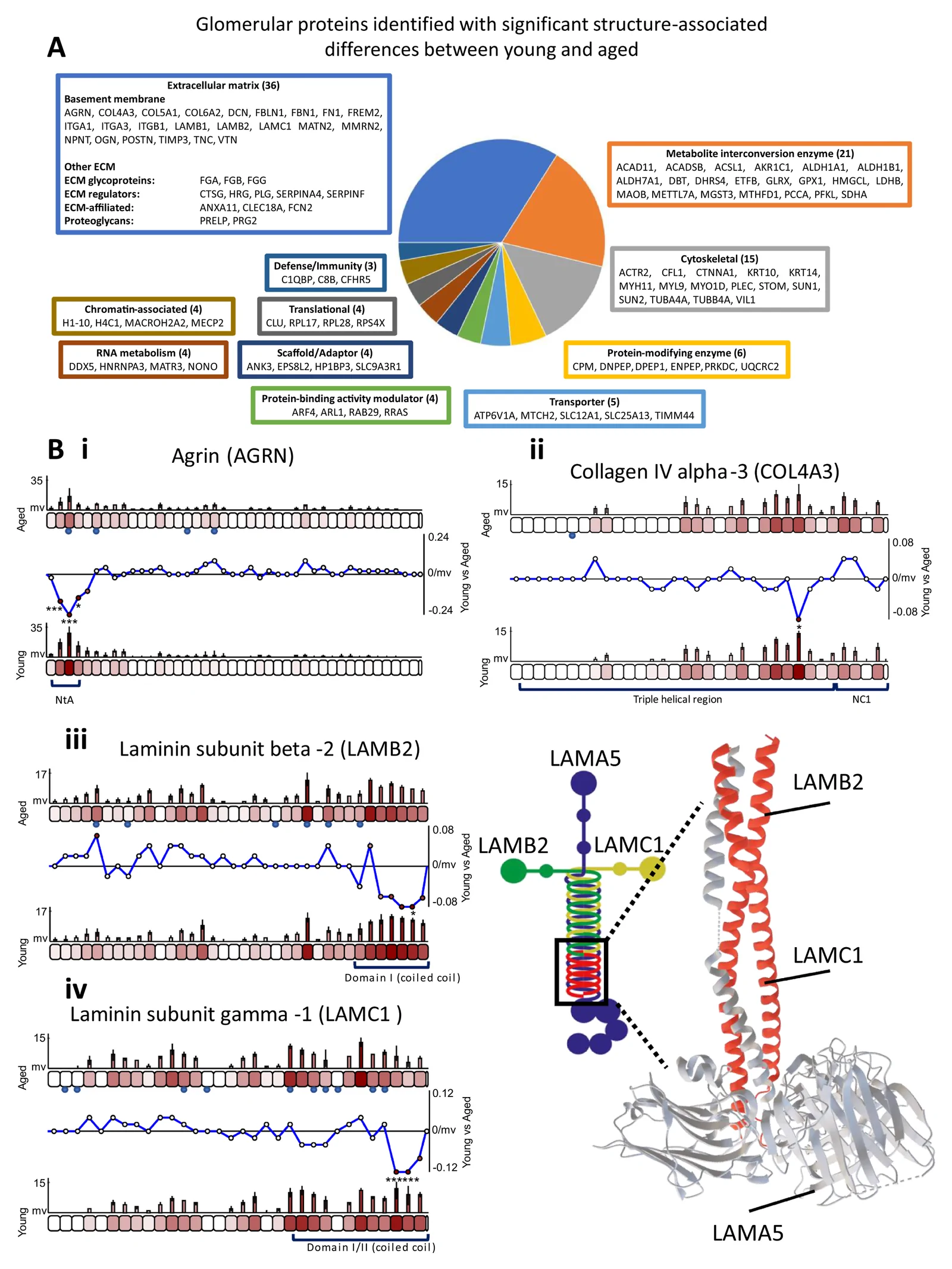
A total of 128 proteins were identified with statistically significant structure-associated differences between young and aged in glomerular samples. Classification analysis (**A**; MatrisomeDB for ECM [29], bmBASE for BM [30], and PANTHER [31] for all others; only classes with ≥ 3 proteins are shown) revealed ECM proteins as the main class affected, 64% of which were BM proteins. Metabolite interconversion enzymes and cytoskeletal proteins were also amongst the top three affected classes. Out of the 23 BM proteins, proteins pertaining to the major GBM components agrin, collagen IV and laminin 521 were further examined. These proteins exhibited significant differences in peptide yield across their structures, which coincided with functional regions **(B)**. During PLF analysis, proteins were segmented into 50 amino acid (aa)-sized segments and proteotypic peptides mapped, quantified (bar graphs = averaged, normalised peptide spectrum matches [PSMs] per segment, error bars = standard deviation [SD], zero values are indicated as missing values [mv]; blue circle = segment contains glycan modification/s [NXT/S triplet; Uniprot sourced]) and statistically tested between young and aged within each (unpaired Bonferroni-corrected, repeated measures ANOVA; *, *p* ≤ 0.05; **, *p* ≤ 0.01; ***, *p* ≤ 0.001). Average peptide yields per segment in young were then subtracted from aged and divided by segment length (50 aa) to reveal differences along protein structure (line graph y axes = aged–young PSMs/segment length). Agrin **(i)** and collagen IV alpha-3 (COL4A3; **ii**) both had protein segments which displayed significantly lower peptide yields in aged than in young that coincided with the N-terminal agrin domain (NtA) and the C-terminal end of the COL4A3 triple helical domain. The laminin chains β2 (LAMB2; **iii)** and γ1 (LAMC1; **iv)** both exhibited significantly lower peptide yields on the C-terminal ends of their coiled-coil protein regions in aged than in young. Laminin 521 (composed of LAMA5, LAMB2 and LAMC1) is the predominating isoform within the GBM of the developed kidney [32]. When considering how LAMB2 and LAMC1 arranges within the laminin 521 quaternary structure, we can see that the age-affected coiled-coil regions on the ends of both proteins overlap within the complex (red on schematic). To aid in these interpretations, protein crystal structures were downloaded from the Protein Data Bank (PBD) and age-affected 50 aa segments were highlighted in red. These affected regions within the coiled coil, together with the LAMA5 globular (G) domains, form part of the integrin-binding region of the laminin complex, as shown by Takizawa et al. (PDB ID: 5XAU) [33].

Proteotypic peptides corresponding to a total of 840 proteins were identified by MS/MS ion searches both in aged and young human glomerulus samples (Table S1; Fig. S1; only proteins which were present in both groups could be interrogated by PLF). Of these, 128 proteins exhibited statistically significant, structure-associated differences between aged and young individuals (Table S2), with ECM-associated proteins, metabolite interconversion enzymes and cytoskeletal proteins being the top three classes affected (Fig. 2A). Crucially, PLF analysis uniquely identified 28 ECM biomarker candidates, which had not been previously identified by whole protein MS1 quantification [11], with significant differences in peptide yield along protein structures but not in relative abundance (Fig. S2). Of the 36 ECM proteins shortlisted by PLF (Fig. 2A), the majority (23) were BM-associated, indicating substantial structural remodelling in aged glomerulus compared to young.

The BM proteins identified with significant structure-associated differences in ageing (Fig. 2A) included several laminin chains, α chains from collagens IV, V and VI, the proteoglycans agrin and mimecan, and several integrin subunits. As major structural components of the GBM, agrin, COL4A3, LAMB2 and LAMC1 all exhibited significant differences in peptide yields along their structures, many of which coincided with biological domains or functional regions (Fig. 2B).

Agrin is the major heparan sulphate (HS) proteoglycan component of glomerulus [34] and contributes heavily to the negative charge state of the GBM due to its anionic glycosaminoglycan side chains [35]. Interestingly, three 50 aa segments specific to the NtA domain of agrin displayed significantly lower peptide yields in aged glomerulus compared to young (Fig. 2B i). To determine whether these NtA-specific differences relate to MMP susceptibility, this region was correlated against predicted MMP cleavage sites derived from the Manchester Proteome Cleave (MPC) deep learning model (applied via the Manchester Proteome Susceptibility Calculator [MPSC] webtool [18]). Interestingly, four predicted MMP7 cleavage sites were identified within two of the age-affected segments (Fig. S3 A ii). Furthermore, the NtA domain of agrin yielded significantly more non-/semi-tryptic peptides (containing possible endogenous cleavages) in young than in aged (Fig. S3 A iii). These observations indicate that heightened MMP-specific fragmentation within this region could be responsible for the greater peptide yields seen in young glomerulus (Fig. 2B i). Patel et al. previously showed *in vitro* that the NtA domain within a recombinant version of agrin is cleavable by MMPs 1, 7, 12 and 14 [36] and that the release of the NtA domain from agrin by MMP12 abolishes laminin interactions. Within the GBM, agrin forms a bridge between the coiled-coil region of laminin 521, via interactions with its NtA domain and cell receptors such as dystoglycans and integrins, through interactions with its C-terminal laminin G-like domains [37]. Therefore, it is possible that age-dependant changes in regional digestibility observed within the NtA domain could reflect a global shift in laminin-agrin interaction as a consequence of altered MMP activity between young and aged.

Of the three collagen IV chains that comprise the GBM’s α3α4α5 hexamer, a single 50 aa segment, found within the C-terminal end of the triple helical region of COL4A3, was identified with significantly lower peptide yields in aged than in young (Fig. 2B ii). Age-dependent differences within this segment could reflect changes in the helical, trimeric association of the three chains, which may also impact the overall integrity of the GBM. Additionally, two RGD binding sites exist in close proximity to this affected segment (6 and 45 amino acids downstream) [38] so differences may be indicative of age-related changes in resident cell interactions.

Importantly, regional differences were also observed within the laminin chains LAMB2 (Fig. 2B iii) and LAMC1 (Fig. 2B iv) which relate to their higher order structures within the laminin 521 complex and associated interactions. These proteins both displayed significantly lower peptide yields in aged than young, in several segments corresponding specifically to the C-terminal end of the coiled coil long arm of laminin 521. The quaternary arrangement and structure of this laminin long arm is crucial to its integrin binding, with the LAMC1 carboxyl tail interacting directly with the integrin adhesion site [33]. The differences observed within this region may therefore provide the molecular basis for an age-dependent disruption in podocyte anchorage within the GBM. Interestingly, the C-terminal end of the LAMB2 coiled coil harbours a cluster of predicted MMP1, 3 and 7 cleavage sites (MPC-derived [18]) located within the first of the five age-affected 50 aa segments (Fig. S3 B i, ii), indicating that fragmentation may play a role in structure-associated differences observed. However, no significant differences were seen in the number of non-/semi-tryptic peptides (which may contain endogenous cleavages) yielded from this region (Fig. S3 B iii).

**Fig. 3:**
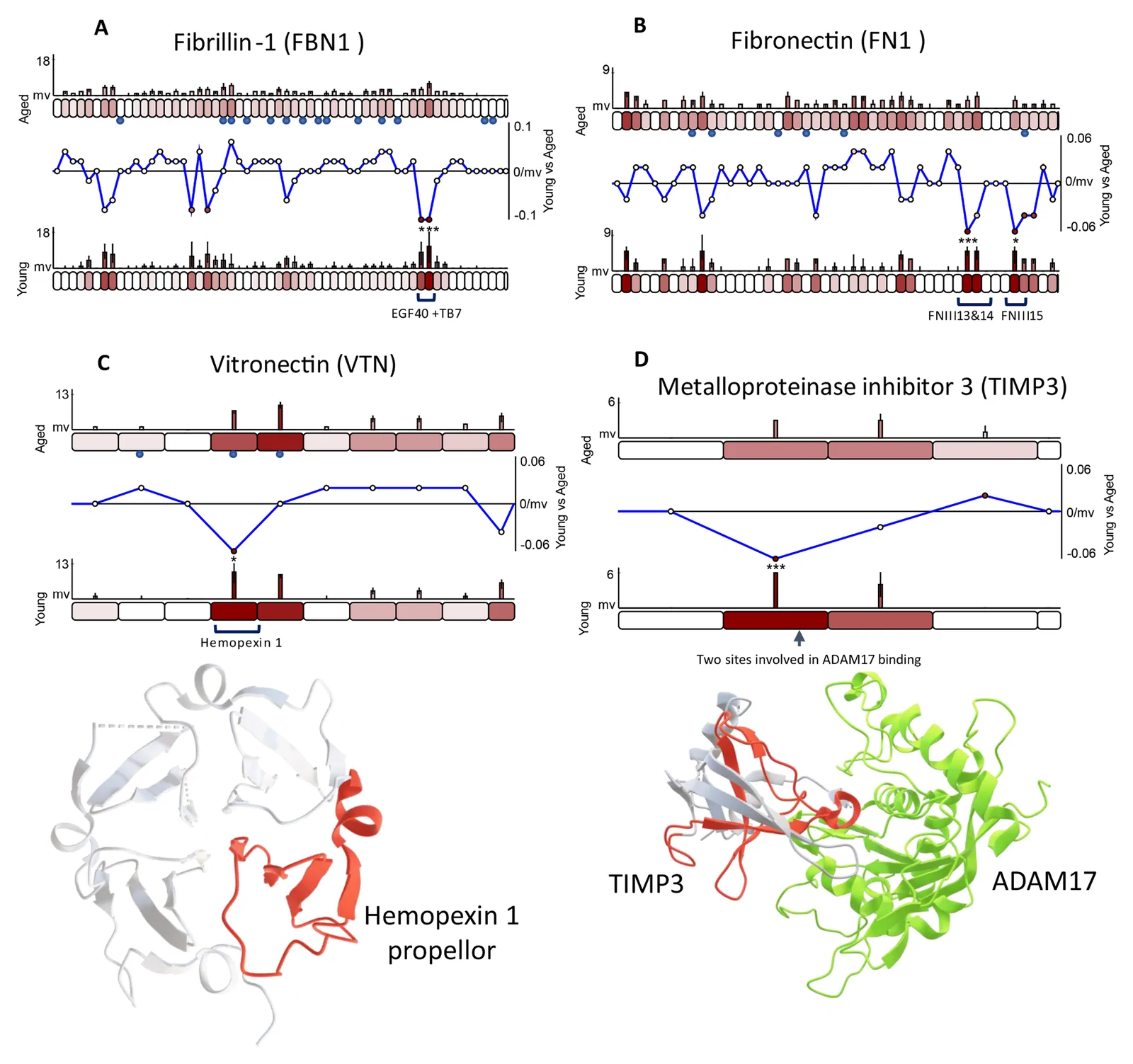
Exemplar ECM proteins of the glomerulus, identified with age-dependant differences in structure which corresponded to important biological domains or functional regions. As in Fig. 2, proteins were segmented into 50 aa-sized segments and proteotypic peptides mapped, quantified (bar graphs = averaged, normalised PSMs per segment, error bars = SD; blue circle = glycan modification/s) and statistically tested (unpaired Bonferroni-corrected, repeated measures ANOVA; *, *p* ≤ 0.05; **, *p* ≤ 0.01; ***, *p* ≤ 0.001) between young and aged. Average peptide yields per segment in young were then subtracted from aged and divided by segment length (50 aa) to reveal differences along protein structure (line graph y axes = aged–young PSMs/segment length). Fibrillin-1 **(A)** and fibronectin **(B)** both had protein regions displaying significantly lower peptide yields in aged than in young. These reside within the epidermal growth factor (EGF) domain 40 and transforming growth factor (TGF)-β binding protein (TB)-like domain 7 of fibrillin 1 and the fibronectin type-III (FNIII) domains 13,14 and 15 of fibronectin. Vitronectin harboured one 50 aa segment near the central portion of its primary structure which displayed lower peptide yields in aged than in young **(C)**. These age-related differences were exclusively found within its hemopexin 1 domain, which together with hemopexins 2–4 form a propellor shaped tertiary structure (PDB ID: 6O5E) with a functional centre capable of binding calcium and heparin [39]. The second 50 aa segment of TIMP3, which also exhibited significantly lower peptide yields in aged compared to young, contains two sites involved in ADAM17 binding **(C)**; better visualised within the crystal structure (PDB ID: 3CKI) of TIMP3 (grey), where the affected segment (red) harbours the docking loop necessary for binding to ADAM17 (green) [40].

In addition to major structural BM components, several other prominent ECM proteins of the glomerulus were identified with significant age-dependent differences in structure (Fig 2A), some of which coincided with important functional regions or biological domains (Fig. 3), including within the elastic fibre protein fibrillin-1, the ECM glycoproteins fibronectin and vitronectin and ECM protease inhibitor TIMP3 (tissue inhibitor of metalloprotease 3).

Fibrillin-1 is the main component of fibrillin microfibrils which, together with elastin, form part of elastic fibres [41]. In the glomerulus however, fibrillin microfibrils are laid down without elastin within the mesangial matrix by resident mesangial cells where they are capable of influencing cell migration and proliferation [42]. Structure-associated differences were observed in segments located in the C-terminal half of fibrillin-1, specifically within the EGF domain 40 and TB domain 7 (Fig. 3A). Previously, the TB7-EGF pair was thought to be a major folding region in a model predicting how fibrillin-1 is organised within the microfibril [43,44]. As such, age-dependent changes in this domain impairing trypsin digestibility, could reflect damage to the fibrillin microfibril ultrastructure (as previously demonstrated by ultraviolet irradiation *in vitro* [24]). Alternatively, the C-terminal half of fibrillin-1 can interact with the proteoglycans neurocan and versican, HS and fibronectin [41], and so peptide yield changes in this region could also reflect dynamic shifts in these interactions during ageing.

Fibronectin is a dimeric glycoprotein which forms fibrillar networks within the pericellular matrix and regulates cell adhesion, migration, proliferation, and differentiation via interactions with multiple integrin receptors [45]. It also exists as a bridge between cells and the wider ECM via multiple collagen- and heparin/HS-binding domains [45]. Fibronectin derived from aged glomerular samples yielded significantly less peptides than from young, in segments residing within the FNIII domains 13,14 and 15 (Fig. 3B) Of these domains, FNIII 13 & 14 contain heparin/HS-binding sites whose interactions appear to be important for fibronectin integration within the wider ECM [46]. In the glomerulus, fibronectin is present in the Bowman’s capsule and the mesangium [47], and genetic variants within its heparin-binding domains (specifically within FNIII 13) have been heavily implicated in the rare autosomal dominant disease, “glomerulopathy with fibronectin deposits” (GFND) [48]. It is possible therefore that the age-related differences identified within this region reflect a change in heparin/HS binding, with potential consequences to glomerular function.

The ECM glycoprotein vitronectin, which has been described as a “biological superglue” crucial to tissue remodelling and repair [49], was also identified with structure-associated differences in ageing. As a matricellular protein, vitronectin serves to interconnect cells, ECM proteins, cytokines, and proteases to facilitate cell surface interactions [49]. Peptide yield differences across the structure of vitronectin were relatively stable between young and aged glomeruli, except for one segment which yielded significantly less peptides in aged (Fig. 3C). Interestingly, this segment falls specifically within one of vitronectin’s four hemopexin domains, each of which forms a blade of a β/α-propellor that is thought to act as a substrate for a myriad of interactions [39] that could therefore be affected in ageing.

Age-associated differences were also observed within a protein region of TIMP3 which contains the docking loop required for ADAM (a disintegrin and metalloproteinase) 17 binding (Fig. 3D) [40]. In addition to the role TIMP3 plays in inhibiting ECM proteolysis [50], this interaction is particularly important for the regulation of inflammation, by inhibiting the ADAM17-mediated activation of tumour necrosis factor α (TNFα) [40]. Disruptions therefore within the TIMP3 protein region that harbours these binding sites could contribute to the TNF-linked “inflammaging” previously described within the kidney [51].

### Tubular basement membranes exhibit structure-associated changes in ageing

**Fig. 4:**
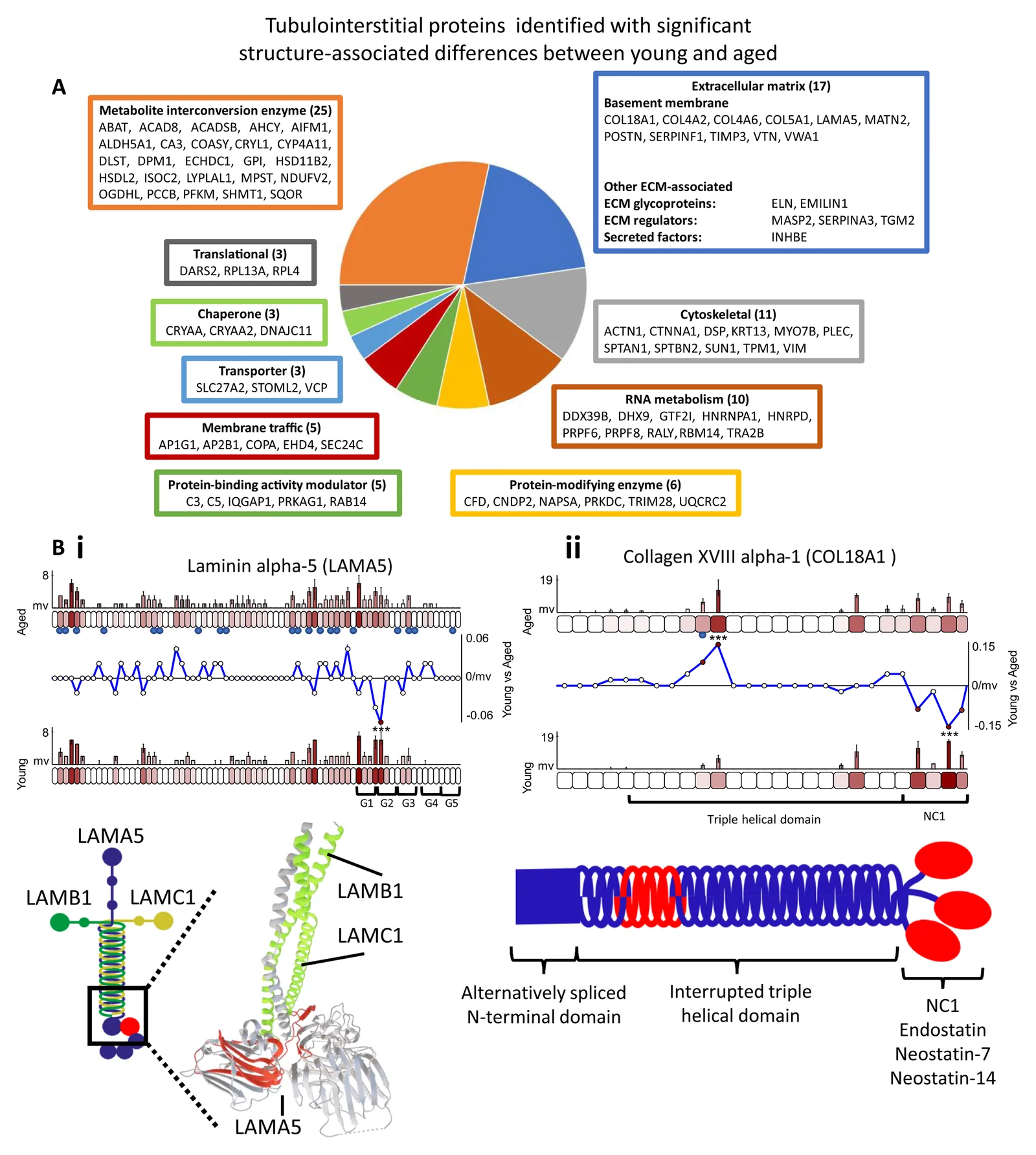
A total of 110 proteins were identified with significant structure-associated differences between young and aged in TBI samples. Classification analysis (**A**; MatrisomeDB for ECM proteins [29], bmBASE for BM [30] and PANTHER [31] for all others; only classes with ≥ 3 proteins are shown) revealed metabolite interconversion proteins as the main class affected, followed by ECM proteins, of which 65% were BM-associated. Of the 11 BM proteins shortlisted, two exemplars are displayed with significant differences in peptide yield across their structures **(B)**. As earlier, proteins were segmented into 50 aa-sized segments and peptides mapped, quantified (bar graphs = averaged, normalised PSMs per segment, error bars = SD; blue circle = glycan modification/s) and statistically tested between young and aged (unpaired Bonferroni-corrected, repeated measures ANOVA; *, *p* ≤ 0.05; **, *p* ≤ 0.01; ***, *p* ≤ 0.001). Average peptide yields per segment in young were then subtracted from aged and divided by segment length (50 aa) to reveal differences along protein structure (line graph y axes = aged–young PSMs/segment length). LAMA5 and collagen XVIII α1 (COL18A1) both had protein regions displaying significantly different peptide yields in aged compared to young. As in the glomerular samples, these affected protein regions coincided with functional, biological domains of their respective ECM assemblies (red in schematics and PDB structure [PDB ID: 5XAU] [33]), as seen in the G2 domain of laminin 511 **(i)**, the N-terminal end of the triple helical domain and in the matrikine-producing NC1 domain of collagen XVIII **(ii)**.

Proteotypic peptides corresponding to 1056 proteins were identified in both young and aged TBI samples by MS/MS ion searches (Table S3; Fig. S4). To test whether tubular BMs also undergo structural remodelling, peptides corresponding to these proteins in glomeruli-depleted TBI samples were also analysed by PLF. A total of 110 proteins were identified with significant differences in peptide yield across their primary structures (Table S4; ~10% of proteins identified by LC-MS/MS). Classification analysis revealed metabolite interconversion enzymes as the main class affected, followed by ECM- and cytoskeletal-associated proteins (Fig. 4A). Crucially, PLF uniquely identified thirteen potential ECM biomarkers of kidney TBI ageing which were not previously identified by standard whole protein MS1 relative quantification (Fig. S5) [11]. Of the seventeen ECM proteins identified with structure-associated differences in ageing, most were BM-specific (11; Fig. 4A) indicating potential remodelling within human kidney tubules.

The BM proteins identified with significant structure-associated differences in ageing (Fig. 4A) included chains from laminin (LAMA5) and collagens IV, V and XVIII as well as the ECM adaptor protein matrilin 2, (whose loss of function was revealed to be associated with BM disease and altered composition [30]). As prominent components of the tubular BM, LAMA5 and COL18A1 are displayed in Fig. 4B as exemplar proteins with significant differences in peptide yields along their structures.

Two neighbouring segments in LAMA5 yielded less peptides in aged than in young TBI (significant in one), corresponding to the globular G2 domain of the laminin long arm. (Fig. 4B i). As previously discussed, the quaternary arrangement and structure of the laminin long arm is crucial to its integrin binding with the G2 domain of LAMA5 interacting directly with this receptor [33]. Therefore, changes within this region may reflect an age-dependent disruption in the epithelial cell anchorage within tubular BMs, similar to that suggested for podocyte anchorage in GBMs (Fig. 2B).

The non-fibrillar collagen XVIII is a common component of vascular and epithelial BMs. Its homotrimeric assembly (composed of COL18A1) exists as three separate splice forms: short, middle and long, and its triple helical domain is interspersed with HS proteoglycan-like features which harbour glycosaminoglycan chains [52]. COL18A1 peptides were matched to the short form of this protein by MS/MS ion searches. Crucially, multiple segments of COL18A1, which lie within the highly functional NC1 domain, yielded lower peptides in aged TBI than in young (Fig. 4B ii). This domain is well known for being cleaved off by cathepsin L [53,54] to release the 20 kDa matrikine fragment endostatin, which can regulate endothelial cell migration, proliferation and apoptosis; thereby acting as a potent inhibitor of angio- and tumori-genesis [55–57]. Additionally, MMP7 and the membrane-bound MMP14 have also been shown to release two alternate bioactive fragments (neostatins-7 and −14 respectively) from this NC1 domain [53, 58], both of which are capable of exerting angiogenic control [59]. In fact, the N-terminal side of the NC1 domain harbours a cluster of predicted (by MPC [18]) and experimentally confirmed [60] MMP1, 3, 7, 8, 9, 12 and 13 cleavage sites (Fig. S6 B), which provides further indication that the differences observed within this region may reflect an age-dependent change in the fragmentation of collagen XVIII, and presence of these matrikines, with functional consequences to tubular BM-epithelial cell communication.

### Periostin and collagen IV α2 exhibit structure-associated differences in ageing that are conserved between human kidney and mouse lung

On an organ-level, BMs in both the kidney glomerulus and lung alveolus share the crucial function of barrier filtration, enabling the selective permeation of small molecules from blood to urine and exchanges of gases respectively [28]. In addition, just like in kidney tubules, BMs in the lung are also found in tubular tissues such as in bronchi and blood vessels. As a result of these shared functions, BMs in both organs harbour many of the same components, such as the α1α1α2 and α3α4α5 hexamer forms of collagen IV, laminin-521, –511 and –111, collagen XVIII, and the proteoglycans agrin, biglycan and decorin [28, 30]. Diseases which specifically target these components can also lead to pulmonary and kidney pathologies, such as in Goodpasture syndrome, where autoantibodies targeting the NC1 domain of COL4A3 in kidneys and lungs induce pulmonary haemorrhage and glomerulonephritis [61]. We propose therefore that certain BM components share similar fates of ageing in both organs, and that their identification could serve as more universal targets for therapeutic intervention.

We recently published the PLF analysis of aged and young mouse bulk lung (Fig. 1) and found that BM proteins were the main ECM components affected by structural modification in ageing [27]. Therefore, to determine whether age-affected BM proteins identified here in human kidney share similar structure-associated consequences in mouse lung, we cross-compared their peptide yield profiles to find matching patterns, which may be indicative of shared mechanisms or consequences of ageing. This comparison also serves to test whether mice (with considerably shorter lifespans than humans) are an adequate model for ageing ECM components, which are known for their longevity.

**Fig. 5:**
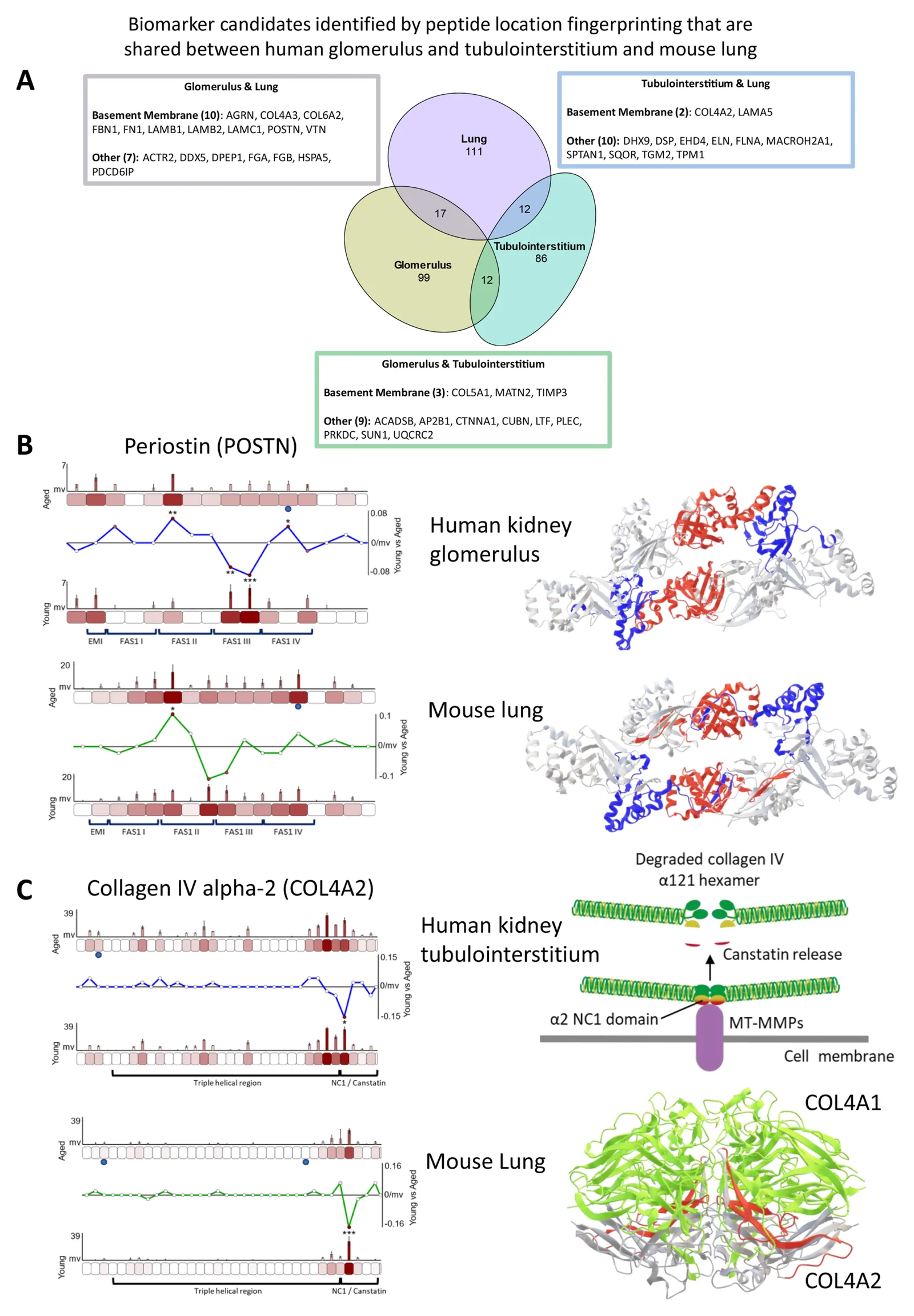
Several BM ageing biomarker candidates identified by PLF were shared between human kidney and mouse lung, with periostin and COL4A2 exhibiting conserved peptide yield differences across structures. A total of 29 proteins were identified with significant structure-associated differences between young and aged, both in human kidney and mouse lung, of which 12 were BM-associated **(A)**. In particular, periostin **(B)** from human kidney glomerulus (blue line) exhibited a very similar peptide yield difference pattern across its protein structure as in mouse lung (green line), with higher peptide yields seen within its second Fasciclin1 (FAS1) domain followed by lower yields near its third FAS1 domain in aged than in young. As earlier, proteins are segmented into 50 aa-sized segments and peptides mapped, quantified (bar graphs = averaged, normalised PSMs per segment, error bars = SD; blue circle = glycan modification/s) and statistically tested between young and aged (unpaired Bonferroni-corrected, repeated measures ANOVA; *, *p* ≤ 0.05; **, *p* ≤ 0.01; ***, *p* ≤ 0.001). Average peptide yields per segment in young were then subtracted from aged and divided by segment length (50 aa) to reveal differences along protein structure (line graph y axes = aged–young PSMs/segment length). This seemingly characteristic rise and fall of peptide yields across the periostin protein can also be visualised along its dimeric higher order structure (PDB ID: 5YJG [62]), with the same affected regions observed in both ageing human kidney glomerulus and mouse lung (red = higher in aged, blue = lower in aged). Similarly, COL4A2 **(C)** from both human kidney TBI (blue line) and mouse lung (green line) exhibited significantly lower peptide yields in aged than in young within the same 50 aa segment located within its globular NC1 domain. The affected segment within the NC1 domain of COL4A2 is susceptible to degradation by membrane-bound MMPs (MT-MMPs) which release canstatin. This segment (coloured red) appears buried within the quaternary structure (PDB ID: 1LI1) of the α1α1α2 hexamer (grey = COL4A2, green = COL4A1).

A total of 140 protein biomarker candidates had been identified with structure-associated differences between aged and young mouse lung [27]. When compared with those identified in human glomerulus (128) and TBI (110), 29 were shared between kidney and lung (Fig. 5A). Of these, 12 were classed as BM proteins, including multiple laminin (LAMA5, LAMB1, LAMB2, and LAMC1) and collagen IV chains (COL4A2 and COL4A3), indicating that many of the same fundamental components of BMs undergo structural remodelling in both lung and kidney ageing.

Periostin and COL4A2 (Fig. 5B, C) both exhibited peptide yield differences between aged and young that were remarkably consistent between human kidney and mouse lung. As a matricellular protein, periostin plays a versatile role in connective tissue maintenance and repair through multiple ECM protein interactions [63]. Although periostin is thought not to be expressed *de novo* in healthy adult kidney [63–65], its presence was identified here in glomerular samples from all young and aged individuals (Tables S1), which may indicate a longevity that is common amongst ECM proteins. Its increased expression in kidney has been directly implicated in kidney injury and chronic kidney disease fibrosis and inflammation [63]. Age-dependent differences in peptide yield were observed within the second and third FAS1 domains in both human kidney and mouse lung (Fig. 5B). These domains are thought to be crucial for the role of periostin in tissue maintenance, repair and disease through their interactions with tenascin C [66], BMP1 [67] (promoting the lysyl oxidase-mediated crosslinking of collagen [68]), CCN3 [69] and cell integrins [70] (stimulating proliferation, survival and matrix deposition pathways [63]). Therefore structure-associated differences within its FAS1 domains may reflect a wider detrimental change in the molecular interaction of periostin across kidney and lung.

Conserved age-dependent changes in collagen IV were also identified in lung and kidney. The same 50 aa segment, near the C-terminal end of COL4A2, exhibited significantly lower peptide yields in both aged human kidney and aged mouse lung, compared to young (Fig. 5C). To determine whether these differences relate to regional oxidation, ROS-sensitive amino acid residues (Trp, Tyr, Met, Cys and His) were quantified across the COL4A2 protein structure (Fig. S7 B i). Interestingly, the NC1 domain harbours ~4.5 times more ROS-susceptible residues than the remaining structure, with the age affected-segment containing the highest content (22%). Additionally, young human TBI samples yielded more peptides identified with oxidised Met (Met-O) from the COL4A2 NC1 domain than aged samples (Fig. S7 B ii). These observations indicate that regional oxidation may be playing a role in the age-dependent differences in peptide yield seen within collagen IV (Fig. 5C). Several studies have shown that the Met-O content of proteins increases with age [71] and the ROS-mediated cross-linking of the collagen IV NC1 domain, and the resulting loss of integrity of the GBM, has been previously proposed [72]. Furthermore, Kalluri et al. showed that ROS-dependent oxidation of collagen IV NC1 hexamers exposes previously cryptic α3 epitopes and enhances binding of Goodpasture syndrome antibodies (including *in vivo*) [73]. These studies highlight that ROS-mediated oxidation of the collagen IV NC1 is likely to be deleterious, however, our results suggest that oxidation within the COL4A2 domain is higher in young TBI BM rather than aged, revealing a potential new mechanism and area of enquiry. Alternatively, the age-affected segment located within the COL4A2 NC1 domain (Fig. 5C) is known to be cleaved by MMP3, MMP9 [74] and MMP15 [75] to release the functional matrikine fragment, known as canstatin [75,76]. Although this region did not harbour a cluster of predicted MMP3 cleavage sites (MPC-derived [18]), one semi-tryptic peptide (AHNQDLGLAGSC) was identified in young TBI samples only, containing an N-terminal cleavage position matching that predicted by MPC for MMP3 (Fig. S7 C i) within the same age-affected segment. The NC1 domain also yielded a higher number of non-/semi-tryptic peptides in young than in aged (Fig. S7 C ii). Since this segment appears buried within the globular NC1 domain of the collagen IV α1α1α2 hexamer [62] (Fig. 5C), and so likely protected from trypsin digestion, these results may be reflective of MMP-mediated canstatin release, which would allow for subsequent trypsinisation. As a matrikine thought to play a major role in tissue homoeostasis through the inhibition of cell apoptosis, proliferation and angiogenesis [76], it is interesting to speculate whether the decreased presence of canstatin in aged lung and kidney may drive inter-tissue disease phenotypes observed in both of these ageing organs.

## Conclusion

Through the application of PLF as a bioinformatic screening tool, we identified ECM proteins with significant structure-associated differences between aged and young kidney, revealing extensive structural remodelling within both glomerular and tubular BMs during ageing. Several of the biomarker candidates shortlisted here had not been previously identified by relative quantification, indicating that the application of PLF in tandem to standard MS1 quantification enables a more complete assessment of tissue proteostasis. Crucially, age-dependent differences were mainly found within functional regions of proteins that display a high level of interaction, reflecting a pervasive disruption in BM proteostasis and its surrounding tissue; as observed in the laminin-binding NtA domain of agrin, the HS-binding FNIII 13 & 14 domains of fibronectin, the integrin-binding C-terminal coiled coil regions and G2 domains of laminin 511/521 and the ADAM17-binding motif of TIMP3.

The key finding that many of the same BM components appear to be structurally affected both in ageing mouse lung and human kidney, with periostin and collagen IV displaying conserved age-dependent differences in structure, points towards the possibility that BM ageing is mediated by mechanisms or consequences that are common across multiple tissues. A more robust identification of these universal targets may prove instrumental to understanding the process of BM ageing and in devising novel therapeutic interventions.

Particular consideration should be given to the collagen IV and collagen XVIII NC1 domains, and their derived matrikine fragments (e.g. canstatin and endostatin), as two PLF studies have now shown changes within this region both in the context of ageing in kidney and lung [27]. The prospect that the NC1 domains of these BM collagens may be particularly susceptible to damage, which may drive tissue degeneration via perturbed matrikine signalling, merits further investigation.

## Materials and methods

### Kidney dataset sourcing and summaries of sample preparation and mass spectrometry methods

Raw LC-MS/MS spectrum files corresponding to ECM-enriched pellet samples were downloaded from the PRIDE data repository (PXD026002) and reprocessed for this study. Sample preparation and mass spectrometry methods pertinent to these datasets are described in Randles et al. 2021 [11]. Briefly, six human kidneys were obtained under approval of the regional ethics committee (05/Q0508/6, University College London; 06/Q1406/38, Manchester) and grouped according to their donor individual ages (young: 15-, 29-, and 37-years old; old: 61-, 67-, and 69-years old). Glomeruli and TBI were isolated through differential sieving and collected in ice-cold PBS. Next, both glomerular and TBI samples were enriched for ECM proteins through chemical fractionation as described previously [77]. For this, samples were treated with an extraction buffer (10 mM Tris, 150 mM NaCl, 25 mM EDTA, 1% Triton X-100, and protease inhibitors) and centrifuged to obtain the first soluble fraction (supernatant), and the remaining pellet was incubated with an alkaline buffer (20 mM Na_4_OH and 0.5% vol/vol Triton X-100 in PBS) and centrifuged to obtain the second soluble fraction (supernatant). The remaining ECM-enriched pellet was then treated with DNAse I and resuspended in a reducing buffer (50 mM Tris-HCl, 10% glycerol, 4% SDS, 0.004% bromophenol blue, 8% β-mercaptoethanol) and heated for 10 min at 95 °C to denature the ECM proteins. Only datasets pertinent to ECM-enriched pellet samples were analysed by PLF in this study, as these were the ones most enriched in basement membrane-specific proteins. Protein samples were submitted to in-gel trypsin digestion as described previously [78] and the resulting peptide samples were processed for tandem mass spectrometry analysis with a nanoACQUITY UltraPerformance liquid chromatography system (Waters) coupled to an LTQ Velos Mass Spectrometer (Thermo Fisher Scientific) or to an Orbitrap Elite analyser (Thermo Fisher Scientific) under data-dependent acquisition mode.

### Peptide identification

Raw LC-MS/MS spectrum files corresponding to ECM-enriched pellet samples were converted to Mascot-compatible peak list files (.mgfs) using RawConverter (Scripps Research Institute, CA, USA) [79]. MS/MS ions were searched against the Uniprot Swiss-Prot and TreEMBL database (2018) [80] using Mascot v 2.5.1 (Matrix Science, MA, USA) with the following parameters – taxonomy: *homo sapiens* (161,629 protein isoforms); fragment tolerance: 0.50 Da; parent tolerance 5.0 ppm (monoisotopic); fixed modifications: + 57 Da on cysteine (carbamidomethyl); variable modifications: +16 Da on lysine, methionine and proline (oxidation) and +42 Da on peptide N-termini (acetylation); digestion enzyme: trypsin; max missed cleavages: 1; peptide charge: 2+ and 3+.

Search result files (.dats) were exported from Mascot and imported into Scaffold 5 (Proteome Software, OR, USA) where peptide spectrum match probabilities were thresholded to ≥ 95%, giving a low peptide false discovery rate (FDR) of 0.6% for both glomerular and TBI datasets. These FDRs were calculated in Scaffold 5 using the PeptideProphet algorithm [81] (WA, USA) based on peptide probabilities assigned by the Trans Proteomic Pipeline. Only peptides exclusive to their matched proteins were selected. Proteotypic peptide lists (Tables S1 and S3) were exported from Scaffold 5 (.csvs) and subsequently analysed by PLF. Spectrum reports, containing peptides identified with fixed and/or variable modifications, were also exported and used to identify Met oxidised peptides.

### Peptide location fingerprinting

PLF was performed for glomerulus and TBI datasets separately using our predeveloped Manchester PLF (MPLF) webtool (accessible at - https://www.manchesterproteome.manchester.ac.uk/#/MPLF) [25], as previously described [25–27]. Peptide lists were imported into the webtool where proteins were automatically segmented into 50 aa-sized segments and peptide sequences and their associated exclusive spectrum counts were mapped and summed within each. To measure regional differences in peptide yield across protein structures that are independent from changes in whole protein abundance, summed peptide counts per segment were median normalised based on their corresponding individual protein total spectrum count. Regional age-associated differences in peptide yield across protein structures were visualised by subtracting the average, normalised peptide counts per segment in the young group from the aged group and dividing by the segment length. Normalised peptide counts per segment were statistically compared between young and aged groups using a Bonferroni-corrected, unpaired repeated measure ANOVA. For more information on PLF and its application, please refer to the original MPLF development publication [25].

### Predicting protein regional MMP and ROS susceptibility

Basement membrane protein regions displaying age-dependent differences in peptide yield were correlated against predicted MMP cleavage sites derived from the Manchester Proteome Cleave (MPC) model (a previously developed [18] deep bidirectional recurrent neural network model capable of predicting cleavage sites for 14 separate tissue proteases), applied via the Manchester Proteome Susceptibility Calculator (MPSC) webtool (accessible at https://www.manchesterproteome.manchester.ac.uk/#/MPSC). As previously described [18], MMP cleavage sites with a high MPC confidence score of ≥ 0.8 were selected within the MPSC webtool for exemplary BM proteins and automatically displayed across their protein structures. Structures were also segmented into 50 aa-sized segments and the number of cleavage sites heat mapped to visualise clustering.

Similarly, the MPSC webtool was used to correlate age-dependent differences across key BM protein structures against predicted ROS-susceptible regions. Within the webtool, ROS-sensitive amino acid (Trp, Tyr, Met, Cys and His) [82] content per 50 aa segment was calculated as a percentage of the total amino acids and automatically displayed as a heat map across the modular protein structure to reveal regional hot spots of susceptibility.

For more information on the MPC prediction model and the MPSC webtool and application, please refer to the original MPSC development publication [18].

### Identification of non/semi tryptic peptides

To identify non/semi tryptic peptides, MS/MS ions were searched against the Uniprot Swiss-Prot and TreEMBL database (2018) [80] using Mascot v 2.5.1 with the same parameters as described in the peptide identification section above but with a non-specific enzyme setting (instead of trypsin). As before, search result files (.dats) were exported from Mascot and imported into Scaffold 5 where peptide spectrum match probabilities were thresholded to ≥ 95%, resulting in a low peptide FDR (Peptide Prophet [81]) of 1.3% for glomerular and 1.4% for TBI datasets. Only peptides exclusive to their matched proteins were selected and peptide lists were exported from Scaffold 5. Non-specific enzyme-matched peptide sequences were filtered against those identified through the trypsin MS/MS ion searches to remove all trypsin-matched peptides from the list. Finally, since trypsin preferentially cleaves at the C-terminal end of Arg and Lys [83], peptides which contained these residues both at the end and just prior to their starting amino acid (except when followed by Pro [83]) were also removed, resulting in a final list enriched in semi/non-tryptic peptides (Table S5 for glomerular; Table S6 for TBI) which could be used for comparison between young and aged.

## Supplementary Material

Supplementary material associated with this article can be found, in the online version, at doi:10.1016/j.matbio.2023.07.001.

Supplementary materials

## Data Availability

The kidney LC-MS/MS datasets analysed by PLF in this paper are available from the PRIDE repository: PXD026002. PLF analyses and associated protein schematics can be interactively viewed and downloaded via the MPLF webtool at https://www.manchesterproteome.manchester.ac.uk/#/MPLF under the “Location Fingerprinter” tab [25,27]. The MPSC webtool [18], used for MMP and ROS susceptibility predictions, is freely available at: https://www.manchesterproteome.manchester.ac.uk/#/MPSC.
