# Supplementary material for: Peptide location fingerprinting identifies structural alterations within basement membrane components in ageing kidney
Source: Matrix Biol. Author manuscript; Available in PMC 2026 Oct 9. (PMC7619537; doi:10.1016/j.matbio.2023.07.001)
Supplement: Supplementary file 1 [file EMS219136-supplement-Supplementary_materials.zip › 1-s2.0-S0945053X23000793-mmc1.pdf]

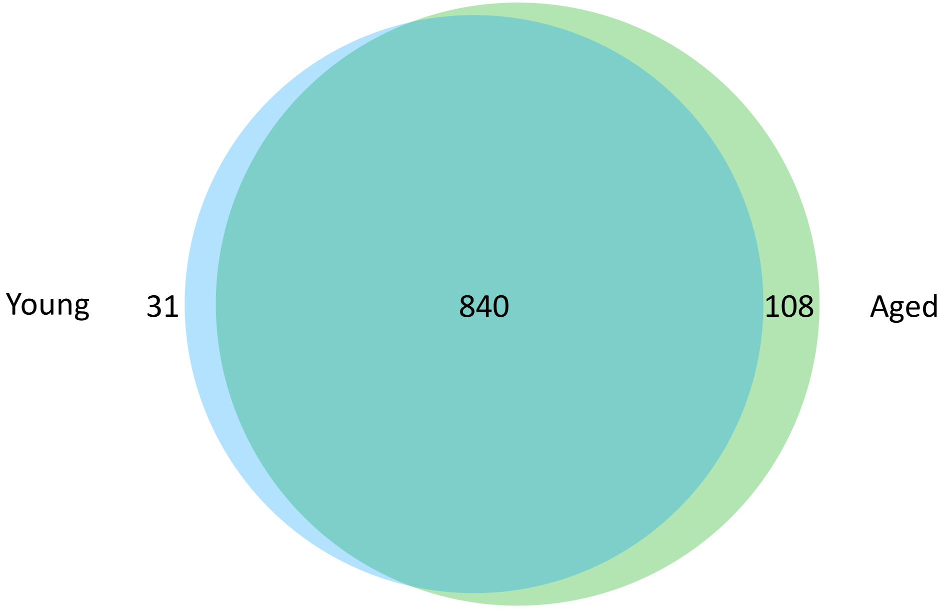

Supporting Figure 1. MS/MS ion searches identified high confident (FDR = 0.6%), proteotypic peptides corresponding to 840 proteins which were shared between young and aged human kidney glomerular samples. These peptides and their associated exclusive spectral counts were then used for PLF analysis.

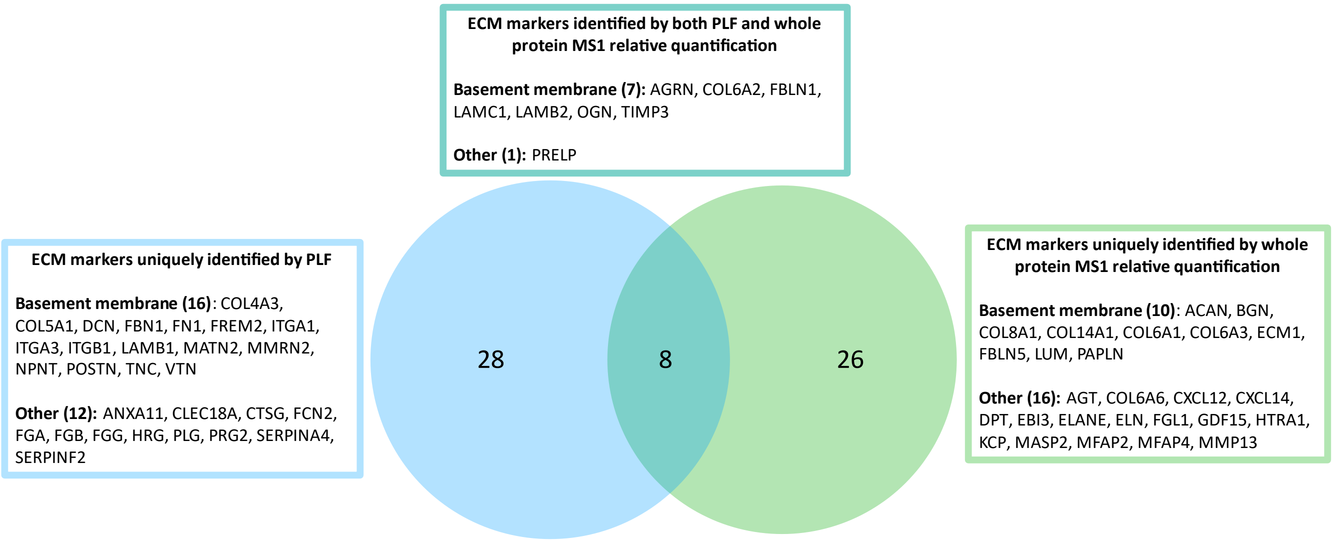

Supporting Figure 2. PLF identified 28 potential ageing ECM biomarkers in kidney glomerulus with significant structure-associated differences in ageing, which were not previously identified by standard whole protein MS1 relative quantification (Randles and Lausecker et al. 2021). Most of these unique markers (57%) were BM proteins (as defined by bmBASE), of which far more were identified with structure-associated differences (16) than solely with differences in relative abundance (10).

Human Glomerulus

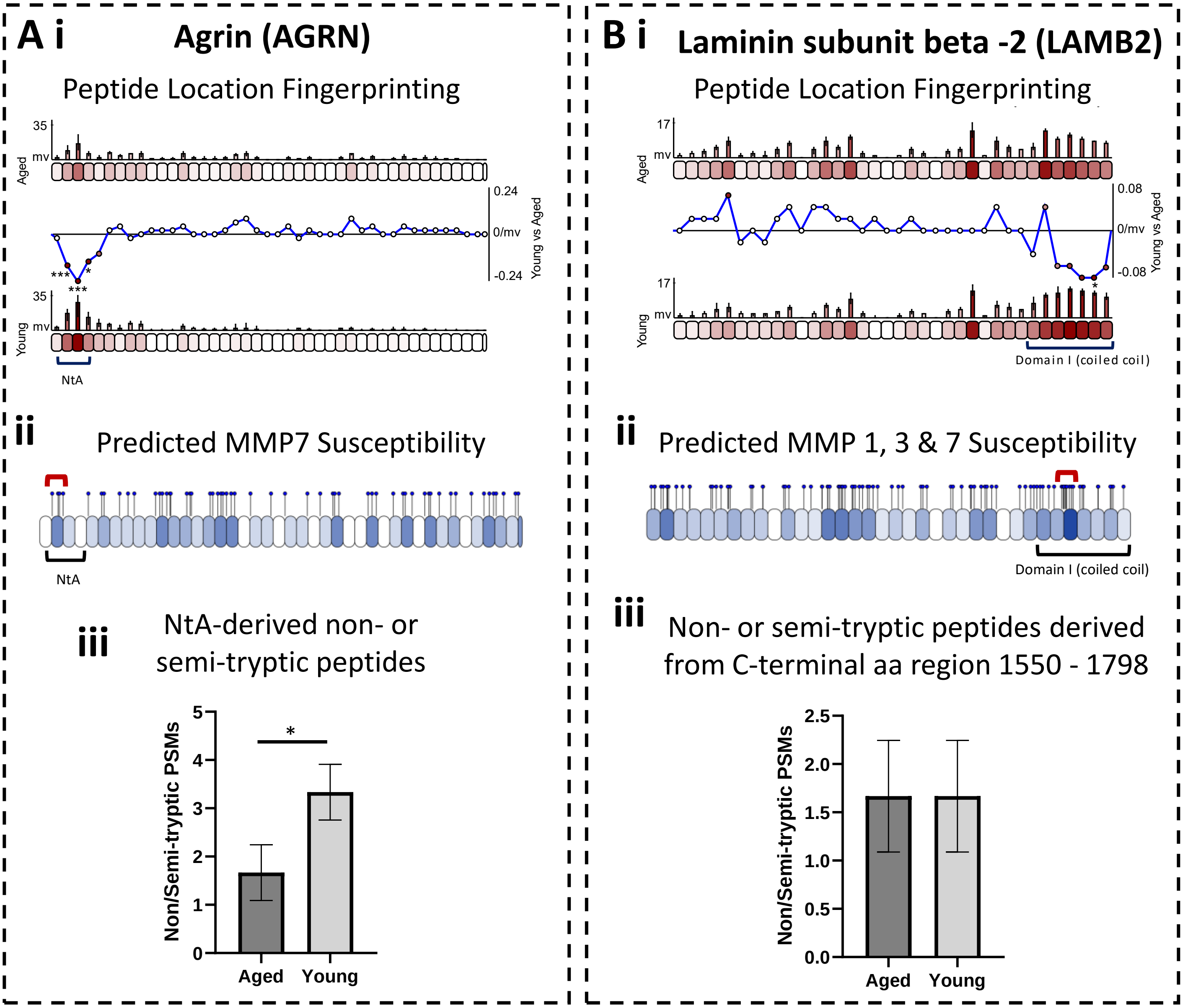

Supporting Figure 3. To determine whether structure-associated differences identified in human glomerular BM proteins agrin and LAMB2 relate to MMP susceptibility, protein regions exhibiting age-dependent differences in peptide yield were correlated against predicted MMP cleavage sites (derived from the Manchester Proteome Cleave [MPC] deep learning model applied via the Manchester Proteome Susceptibility Calculator [MPSC] webtool [Ozols *et al.* 2021]; only cleavage sites with an MPC confidence score of ≥ 0.8 were considered), and non-/semi-tryptic peptides (containing possible endogenous cleavages) were relatively quantified between young and aged (peptide spectrum matches). PLF revealed significantly lower tryptic peptide yields within the NtA region of the proteoglycan agrin derived from aged glomerulus compared to young (**A i;** see main Fig 2B for stats). This same region harbours four predicted MMP7 cleavage sites (red bracket) found directly within the same two age-affected 50 aa segments identified by PLF **(A ii**; blue heat map scales with number of cleavage sites). Furthermore, the NtA region of agrin yielded significantly more non-/semi-tryptic peptides in young than aged (q=0.024, FDR-corrected [Benjamini, Krieger and Yekuteli] unpaired t test), which may indicate higher endogenous cleavage activity. Similarly, the C-terminal region of LAMB2, located within Domain I the laminin long arm, also yielded less tryptic peptides in aged than young **(B i)**. Interestingly, this same region of LAMB2 contains a hotspot of predicted MMP cleavage sites (**B ii**, red bracket), located within the first of the five age-affected 50 aa segments, indicating that fragmentation may play a role in this structure-associated difference between young and aged. However, no significant differences were seen between young and aged in the number of non-/semi-tryptic peptides yielded from this region **(B iii)**.

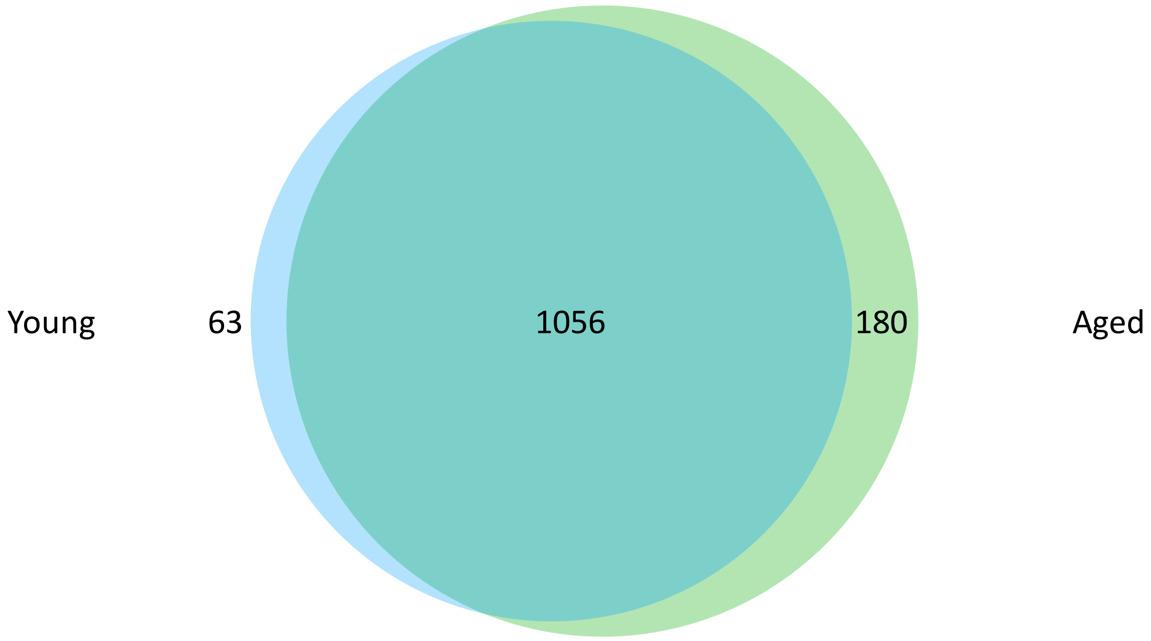

Supporting Figure 4. MS/MS ion searches identified high confident (FDR = 0.6%), proteotypic peptides corresponding to 1056 proteins which were shared between young and aged human kidney tubulointerstitial samples. These peptides and their associated exclusive spectral counts were then used for PLF analysis.

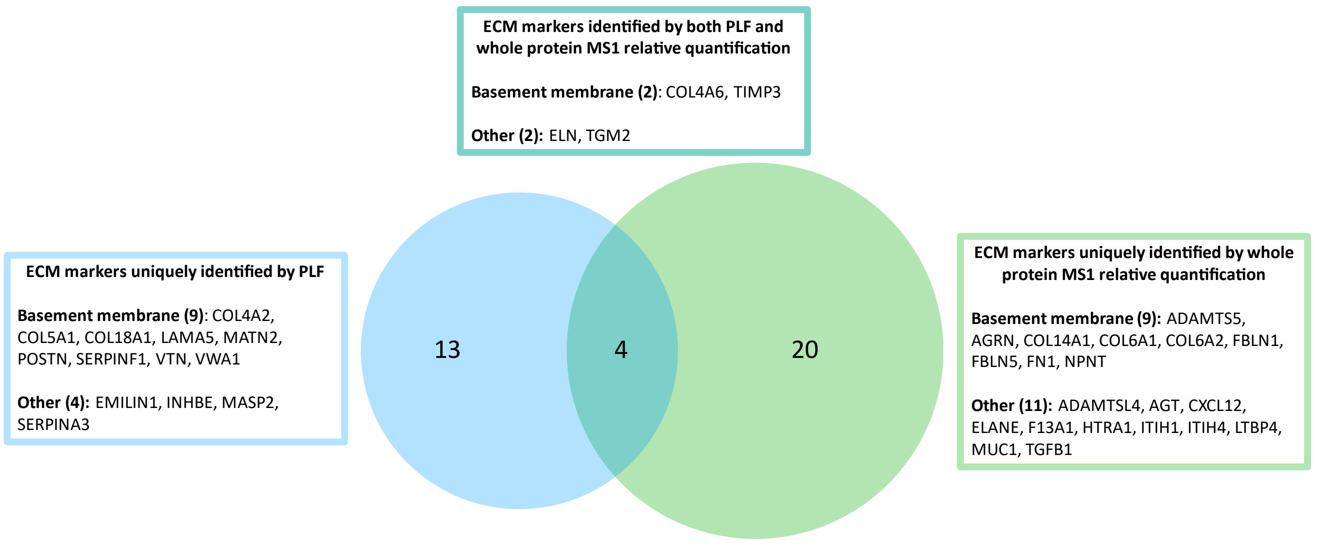

Supporting Figure 5. PLF identified 13 potential ageing ECM biomarkers in kidney tubulointerstitium with significant structure-associated differences in ageing, which were not previously identified by standard whole protein MS1 relative quantification (Randles and Lausecker et al. 2021). As with the glomerular analysis, most of these unique markers (69%) were BM proteins (as defined by bmBASE).

Human Tubulointerstitial
Collagen XVIII alpha-2 (COL18A1)

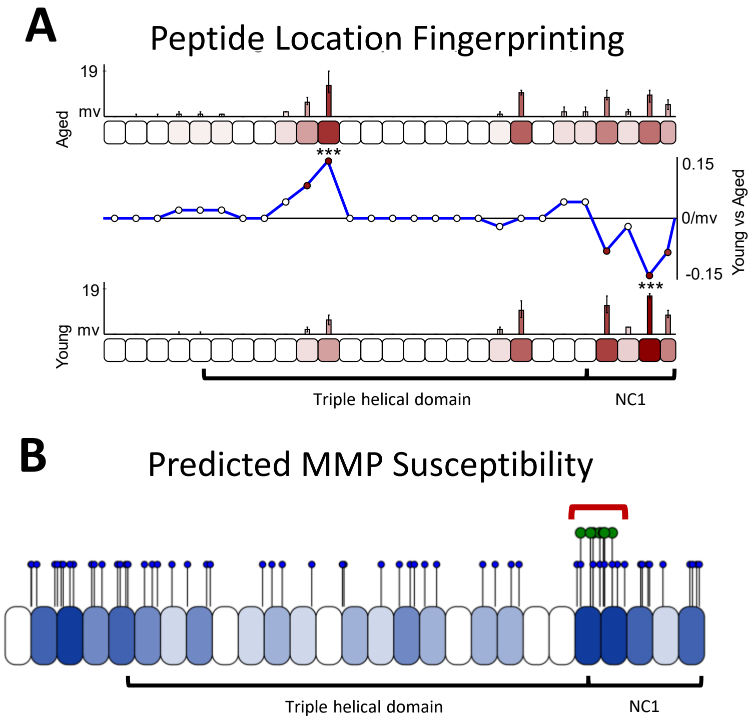

Supporting Figure 6. To determine whether structure-associated differences observed in ageing human tubulointerstitial COL18A1 relate to MMP susceptibility, protein regions exhibiting age-dependent differences in peptide yield were correlated against predicted MMP cleavage sites (MMPs 1, 3, 7, 8, 9, 12, and 13 derived from the MPC deep learning model applied via the MPSC webtool (Ozols *et al*. 2021); only cleavage sites with an MPSC confidence score of ≥ 0.8 were considered). Significantly lower tryptic peptide yields were identified by PLF within the C-terminal NC1 region of COL18A1 in aged compared to young tubulointerstitial samples (**A**; see main Fig 4B for stats). This same region of COL18A1 was found to contain a hotspot (red bracket) of predicted (blue pins) and experimentally validated (green pins; Heljasvaara *et al.,* 2005) MMP cleavage sites (**B**), located within the first two segments of the NC1 region, which indicates that fragmentation may play a role in the structure-associated differences seen in young compared to aged (blue heat map scales with number of cleavage sites).

Human Tubulointerstitial Collagen IV alpha-2 (COL4A2)

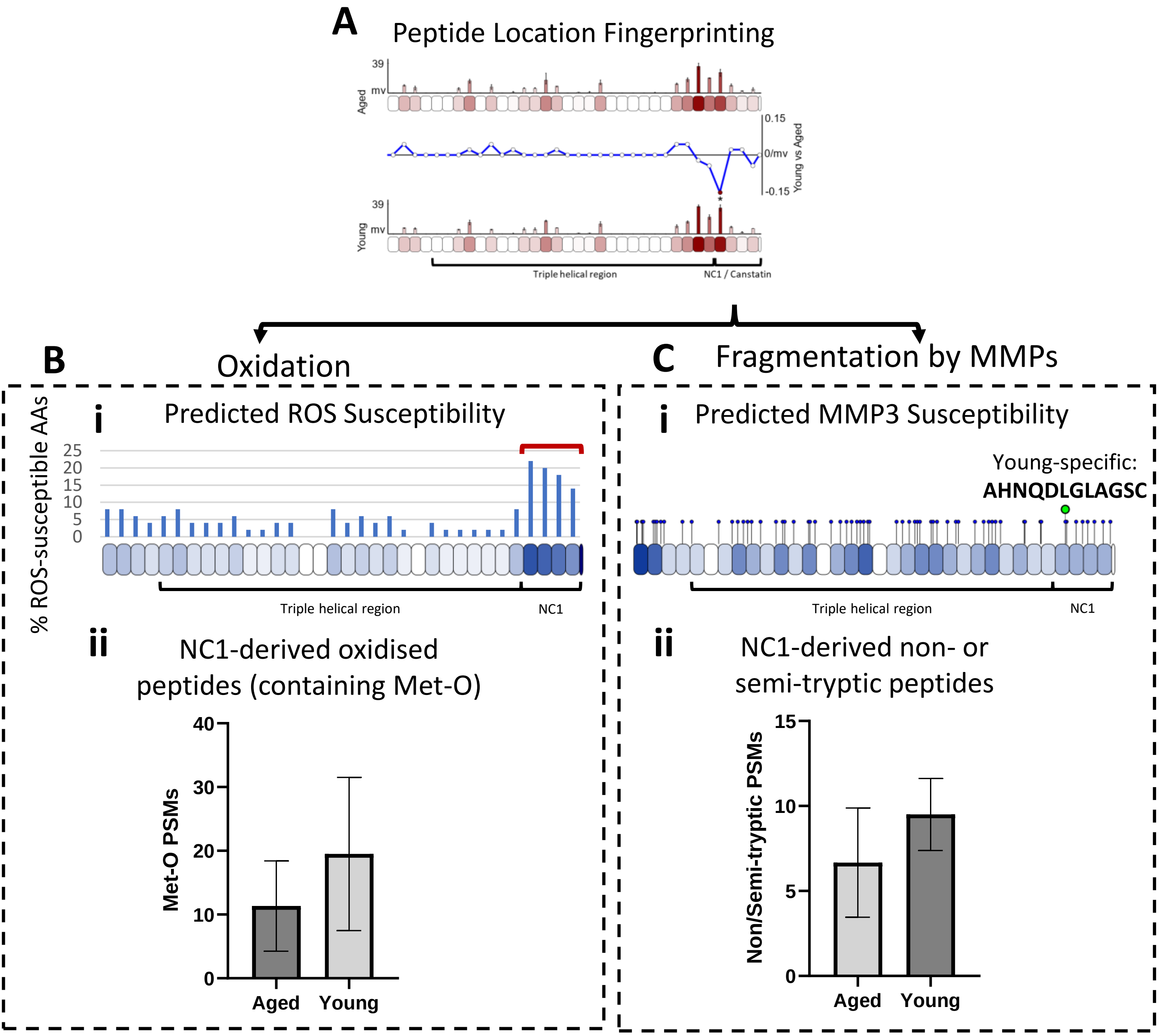

Supporting Figure 7. PLF revealed significantly lower tryptic peptide yields within a 50 aa segment, located on the N-terminal side of the NC1 domain of COL4A2 derived from aged tubulointerstitial samples compared to young (**A**; for stats see main Fig. 5). To determine whether these differences relate to regional oxidation susceptibility across COL4A2 protein structure **(B)**, the percentage of reactive oxygen species (ROS)-sensitive aa residues (Σ[Trp, Tyr, Met, Cys and His]/50*100) was determined per segment using the MPSC webtool **(i)** (Ozols *et al.* 2021) and oxidised peptides (identified with oxidised methionine [MetO]) were relatively quantified between young and aged (**ii**, peptide spectrum matches). Interestingly, the NC1 domain of COL4A2 harbours a concentration (red bracket) of ROS-sensitive residues (14-22%), when compared to the rest of its protein structure. The NC1 domain also yielded more oxidised peptides in young compared to aged COL4A2 (not significant). To determine whether age-associated differences identified by PLF within the NC1 domain relate to MMP3 susceptibility **(C)**, COL4A2 protein regions exhibiting age-dependent differences in peptide yield were correlated with predicted MMP3 cleavage sites (**i**; derived from the MPC deep learning model applied via the MPSC webtool [Ozols *et al.* 2021]; only cleavage sites with an MPSC confidence score of ≥ 0.8 were considered) and non-/semi-tryptic peptides (containing possible endogenous cleavages) were relatively quantified and compared between young and aged (**ii**; peptide spectrum matches). Although this region did not harbour a higher number of predicted MMP3 cleavage sites (blue pins) compared to the remaining protein structure, one semi-tryptic peptide (AHNQDLGLAGSC) was identified in young samples only, containing an N-terminal cleavage position matching that predicted by MPC for MMP3 (green pin). This potential MMP3 cleavage site is found within the same segment which yielded significantly lower tryptic peptides in aged (PLF). Interestingly, a higher number of non-/semi-tryptic peptides were also yielded from the NC1 domain of COL4A2 (though not significant).
